# Supplementary material for: ‘Shape-Up’, a Modified Cognitive-Behavioural Community Programme for Weight Management: Real-World Evaluation as an Approach for Delivering Public Health Goals
Source: Nutrients. 2021 Aug 16;13(8):2807. doi: 10.3390/nu13082807 (PMC8402118; doi:10.3390/nu13082807)
Supplement: Supplementary file 1 [file nutrients-13-02807-s001.zip › nutrients-1306598-supplementary.pdf]

Supplementary file

Table S1: RE-AIM Dimension and Evaluation Criteria Reported in Systematic Review of RE-AIM use

**Reach**	**1**	Exclusion criteria (% excluded or characteristics)
	**2**	Percentage of individuals who participate, based on valid denominator
	**3**	Characteristics of participants compared with nonparticipants; to local sample
	**4**	Use of qualitative methods to understand recruitment
**Effectiveness**	**1**	Measure of primary outcome
	**2**	Measure of primary outcome relative to public health goal
	**3**	Measure of broader outcomes or use of multiple criteria (e.g., measure of quality of life or potential negative outcome)
	**4**	Measure of robustness across subgroups (e.g., moderation analyses)
	**5**	Measure of short-term attrition (%) and differential rates by patient characteristics or treatment group
	**6**	Use of qualitative methods/data to understand outcomes
**Adoption (setting level)**	**1**	Setting exclusions (% or reasons or both)
	**2**	Percentage of settings approached that participate (valid denominator)
	**3**	Characteristics of settings participating (both comparison and intervention) compared with either (1) nonparticipants or (2) some relevant resource data

	**4**	Use of qualitative methods to understand setting level adoption
**Adoption (staff level)**	**1**	Staff exclusions (% or reasons or both)
	**2**	Percentage of staff offered who participate
	**3**	Characteristics of participating vs non participating staff
	**4**	Use of qualitative methods to understand staff participation/staff level adoption.
**Implementation**	**1**	Percent of perfect delivery or calls completed (e.g., fidelity)
	**2**	Adaptations made to intervention during study (not fidelity)
	**3**	Cost of intervention—time
	**4**	Cost of intervention—money
	**5**	Consistency of implementation across staff/time/settings/subgroups (not about differential outcomes, but process)
	**6**	Use of qualitative methods to understand implementation
**Maintenance (Individual Level)**	**1**	Measure of primary outcome (with comparison with a public health goal) at ≥ 6 mo follow-up after final treatment contact
	**2**	Measure of primary outcome ≥ 6 mo follow-up after final treatment contact
	**3**	Measure of broader outcomes (e.g., measure of quality of life or potential negative outcome) or use of multiple criteria at follow-up

	**4**	Robustness data—something about subgroup effects over the long-term
	**5**	Measure of long-term attrition (%) and differential rates by patient characteristics or treatment condition
	**6**	Use of qualitative methods data to understand long-term effects
**Maintenance Setting Level**	**1**	If program is still ongoing at ≥ 6 mo posttreatment follow-up
	**2**	If and how program was adapted long-term (which elements retained after program completed)
	**3**	Some measure/discussion of alignment to organization mission or sustainability of business model
	**4**	Use of qualitative methods data to understand setting level institutionalization

Taken from: *Gaglio, B.; Shoup, J.A.; Glasgow, R.E. The RE-AIM Framework: A Systematic Review of Use Over Time. American Journal of Public Health* ***2013****, 103, e38-e46, doi:10.2105/AJPH.2013.301299.*

JUST STARTING
SHAPE-UP SURVEY

**Your Name:**	
**Today's Date:**	
**E-mail Address:**	
**Date of Birth:**	

How many servings of fruit and vegetables do you typically eat per day? Tick one box. (Fruit and vegetables can be fresh, frozen, canned or dried and one portion is roughly a handful.)

Less than one per day	1	2	3	4	5	6 or more

How often over the past *week* did you do a total of *30 minutes* of physical activity that made you feel *warm and breathe more heavily*? Tick one box.

5 times or more	3 to 4 times	Twice	Once	Never

Please answer the following section by ticking one box for each statement to show which best describes your current lifestyle and behaviours.

1	I have a regular pattern of eating (i.e. roughly eating at the same times each day).	**Strongly agree**	**Agree**	**Not sure**	**Disagree**	**Strongly disagree**

WEIGHTCONCERN
FIGHTING OBESITY WITH KNOWLEDGE

© Weight Concern, 2013. This material can be reproduced for use with Shape-Up Groups.
www.weightconcern.org.uk email: enquiries@weightconcern.org.uk

2	I always have a balance of different types of food in my diet (i.e. lots of starchy foods and fruit and vegetables, and only tiny amounts of fatty/sugary foods and drinks).	Strongly agree	Agree	Not sure	Disagree	Strongly disagree
3	I set effective lifestyle goals and work towards them.	Strongly agree	Agree	Not sure	Disagree	Strongly disagree
4	I am in control of my food portion sizes.	Strongly agree	Agree	Not sure	Disagree	Strongly disagree
5	I know what appropriate food portion sizes are for losing weight.	Strongly agree	Agree	Not sure	Disagree	Strongly disagree
6	I am able to manage triggers that lead to unhealthy behaviours (may include things like your mood, or the sight/smell of tempting food).	Strongly agree	Agree	Not sure	Disagree	Strongly disagree
7	I am confident that I understand the information on food labels.	Strongly agree	Agree	Not sure	Disagree	Strongly disagree
8	I feel in control of my eating habits.	Strongly agree	Agree	Not sure	Disagree	Strongly disagree
9	I feel confident amd motivated to make any lifestyle changes.	Strongly agree	Agree	Not sure	Disagree	Strongly disagree
10	I am happy with my current weight.	Strongly agree	Agree	Not sure	Disagree	Strongly disagree

© Weight Concern, 2013. This material can be reproduced for use with Shape-Up Groups.
www.weightconcern.org.uk email: enquiries@weightconcern.org.uk

Just Starting Weight Record

Your height: ☐ cm

OR ☐ feet and inches

Your weight: ☐ kg (to nearest 0.1kg)

OR ☐ (stones and pounds)

Are you male or female? (Please tick.)

Female ☐ Male ☐

What is the highest qualification you have passed? (Please tick one.)

Degree or equivalent, or higher degree	☐
Non-degree teaching or other professional qualification	☐
'A' level or equivalent	☐
GCSE, 'O' level or equivalent	☐
CSE or equivalent	☐
Other qualifications	☐
No qualifications	☐

Which best describes your ethnic background?

White

British ☐

Irish ☐

Any other white background (please write in): ______________________

Mixed

White and Black Caribbean ☐

White and Black African ☐

White and Asian ☐

Any other mixed background (please write in): ______________________

Asian or Asian British

Indian ☐

Pakistani ☐

Bangladeshi ☐

Any other Asian background (please write in): ______________________

WEIGHTCONCERN

FIGHTING OBESITY WITH KNOWLEDGE

© Weight Concern, 2013. This material can be reproduced for use with Shape-Up Groups.
www.weightconcern.org.uk email: enquiries@weightconcern.org.uk

Black or Black British

Caribbean ☐

African ☐

Any other Black background (please write in): ______________________

Chinese or other ethnic group

Chinese ☐

Any other background (please write in): ______________________

Service evaluation of *Shape-Up*: Informed Consent Form

Please complete this form after you have read the Information Sheet and/or listened to an explanation about the evaluation.

If you have any questions arising from the Information Sheet or the explanation given to you, please ask the group leader before you to decide whether to join in.

Participant's Statement:

I, ______________________

1. have read the notes written above and the Information Sheet, and understand what the service evaluation of *Shape-Up* evaluation.
2. understand that if I decide at any time that I no longer wish to take part in this evaluation, I can notify the service delivery team and withdraw immediately.
3. consent to the processing of my personal information for the purposes of this service evaluation and understand that no individuals will be identifiable from the information when it is shared with Weight Concern.
4. understand that all information will be treated as strictly confidential and handled in accordance with the provisions of the Data Protection Act 1998.
5. understand that the information I have submitted will contribute to the on-going evaluation of the *Shape-Up* programme by Weight Concern. Confidentiality will be maintained at all times and it will not be possible to identify any individual from publications.
6. agree that this evaluation has been explained to me to my satisfaction and I agree to take part.

**Name:**	
**Signature:**	
**Date:**	

If you have any unanswered questions about the service evaluation of *Shape-Up* please feel free to contact Weight Concern on enquiries@weightconcern.org.uk or on 020 7679 1853.

WEIGHTCONCERN

© Weight Concern, 2013. This material can be reproduced for use with Shape-Up Groups.
www.weightconcern.org.uk email: enquiries@weightconcern.org.uk

Question on vigorous exercise added by PfP:

How often over the past week did you do a total of 20 minutes of physical activity that you were sweating and you were breathing hard and fast and your heart rate had increased significantly. If you're working at this level, you won't be able to say more than a few words without pausing for a breath?

5x per week or more	3-4x per week	2x per week	Once	Never
☐	☐	☐	☐	☐

Figure S1. Just starting survey from Shape-Up.

Table S2: Recruitment and flow through the programme

Information supplied by Contracts Health & Wellbeing Manager (Rotherham & Sheffield) (email, 10th July 2019) for Cohort 3.4. The final wave of PfP Shape-Up participants

**Contacts Generated**	377
**Sign Ups**	178
**Did Not Attend (DNA)**	21
**Starters**	157
**Drop Outs**	20
**Completers**	137
**Total Number lost 3-5%**	96
**Completed**	87.26%
**% Completers losing 3-5%**	70%

(NB/ this table includes data from the whole of the wave, which includes participants from four leisure centres, note that only three of these centres were included in the current service evaluation)

Table S3: Quality of life as measured using the EQ-5D-3L, at baseline, endpoint and modal change values

*Scoring- 1, no limitations; 2, some limitations; 3, severe limitations; decrease, improvement; increase, deterioration.

EQ-5D-3L*	Baseline	
	N	%
**QoL Mobility**	**126**	
1	89	71%
2	37	29%
3	0	0%
**QoL Self-Care**	**127**	
1	113	89%
2	12	9%
3	1	1%
**QoL Usual Activities**	**127**	
1	92	72%
2	34	27%
3	1	1%
**QoL Pain/ Discomfort**	**124**	
1	62	50%
2	50	40%
3	12	10%
**QoL Anxiety/ Depression**	**128**	
1	76	59%
2	45	35%
3	7	6%
	**N (%)**	**Modal change**
**QoL Mobility**	**73**	Modal category change = 0 (84.3%)
1	51 (69.9%)	
2	22 (30.1%)	-2 categories (1.4%); -1 category (8.6%);
3	0 (0%)	+1 category (4.3%); +2 category (1.4%)
**QoL Self-Care**	**73**	Modal category change = 0 (84.5%).
1	65 (89.0%)	
2	8 (11%)	-2 categories (0%); -1 category (5.7%);
3	1 (0%)	+1 category (2.8%); +2 category (1.4%)
**QoL Usual Activities**	**73**	Modal category change = 0 (73.2%)
1	52 (71.4%)	
2	20 (27.4%)	-2 categories (2.8%); -1 category (12.7%);
3	1 (1.4%)	+1 category (8.5%); +2 category (2.8%)
**QoL Pain/ Discomfort**	**74**	Modal category change = 0 (69.2%)
**1**	37 (50.0%)	-2 categories (0%); -1 category (13.8%);
**2**	30 (40.5%)	+1 category (13.8%); +2 category (3.1%)
**3**	7 (9.5%)	
**QoL Anxiety/ Depression**	**74**	Modal category change = 0 (66.7%)
**1**	48 (64.9%)	
**2**	18 (24.3%)	-2 categories (2.8%); -1 category (16.7%);
**3**	8 (10.8%)	+1 category (12.5%); +2 category (1.4%)
